# Modeling anti-IL-6 therapy using breast cancer patient-derived xenografts

**DOI:** 10.18632/oncotarget.11815

**Published:** 2016-09-01

**Authors:** Beatriz Morancho, Mariano Zacarías-Fluck, Antonio Esgueva, Cristina Bernadó-Morales, Serena Di Cosimo, Aleix Prat, Javier Cortés, Joaquín Arribas, Isabel T. Rubio

**Affiliations:** ^1^ Preclinical and Clinical Research Programs, Vall d'Hebron Institute of Oncology, Barcelona, Spain; ^2^ Breast Surgical Unit, Breast Cancer Center, Hospital Universitario Vall d'Hebron, Universitat Autònoma de Barcelona, Barcelona, Spain; ^3^ Division of Oncology, Fondazione IRCCS Istituto Nazionale dei Tumori, Milano, Italy; ^4^ Department of Medical Oncology, Hospital Clínic of Barcelona, Barcelona, Spain; ^5^ Translational Genomics and Targeted Therapeutics in Solid Tumors, August Pi i Sunyer Biomedical Research Institute, Barcelona, Spain; ^6^ Department of Biochemistry and Molecular Biology, Universitat Autonoma de Barcelona, Campus de la UAB, Bellaterra, Spain; ^7^ Institució Catalana de Recerca i Estudis Avançats, Barcelona, Spain

**Keywords:** breast cancer, IL-6, STAT3, patient-derived xenografts

## Abstract

The pleiotropic cytokine IL-6 accelerates the progression of breast cancer in a variety of preclinical models through the activation of the STAT3 (signal transducer and activator of transcription 3) signaling pathway. However, the proportion of breast cancers sensitive to anti-IL-6 therapies is not known. This study evaluates the efficacy of anti-IL-6 therapies using breast cancer patient derived xenografts (PDXs). During the generation of our collection of PDXs, we showed that the successful engraftment of tumor tissue in immunodeficient mice correlates with bad prognosis. Four PDXs out of six were resistant to anti-IL-6 therapies and the expression of IL-6, its receptor or the levels of phospho-STAT3 (the active form of the signal transducer) did not correlate with sensitivity. Using cell cultures established from the PDXs as well as samples from *in vivo* treatments, we showed that only tumors in which the activation of STAT3 depends on IL-6 respond to the blocking antibodies. Our results indicate that only a fraction of breast tumors are responsive to anti-IL-6 therapies. In order to identify responsive tumors, a functional assay to determine the dependence of STAT3 activation on IL-6 should be performed.

## INTRODUCTION

In women, breast cancer is the most frequently diagnosed type of cancer and the second leading cause of cancer-related deaths. Despite appropriate adjuvant systemic therapies, up to 30% of patients will relapse. Tumors resistant to the first line of therapy are treated with multiple lines of therapy, in general with little success. Many new drugs are currently being developed to treat these recurrent breast cancers but, giving the numerous failures in the clinic of therapies that show efficacy in laboratory models, there is a pressing need for preclinical models as similar as possible to the real mammary tumor.

In the last decades it has become apparent that breast tumors are composed of different cell populations. This cellular heterogeneity has been proposed responsible for recurrence and resistance to most therapies. Historically, preclinical research relied on cell lines established from tumor specimens. However, cell lines do not reflect breast cancer heterogeneity because they are frequently oligoclonal and, since they propagate as monolayers in vitro, in conditions that differ greatly from those in the tumor, they are arbitrarily selected. In the 1980s, xenografted human tumor models, also known as patient-derived xenografts (PDXs), emerged as an experimental system with many advantages over established cells lines. Tumor xenografts frequently maintain the cell differentiation and morphology, the architecture, molecular signatures and intratumoral heterogeneity of the original tumor and are considered by several authors predictive preclinical models [1-6].

Interleukin-6 (IL-6) is a multifunctional cytokine originally described as a regulator of the immune and inflammatory responses. IL-6 is presented by its specific receptor (IL-6 receptor alpha, IL6RA) to a signaling receptor (gp130), which is shared by other cytokines [7]. The complex IL-6/IL6RA/gp130 activates different intracellular signaling pathways, the most prominent of which is the JAK (Janus kinases)-STAT (signal transducer and activator of transcription) pathway. Of the members of the STAT family, STAT3 has been shown to be the most important for breast cancer progression [8].

The expression of IL-6 is elevated in different tumors of epithelial origin [9]. In breast cancer patients, the increase in serum IL-6 correlates with poor disease outcome and prognosis [10]. Accordingly, blockade of IL-6 signaling, through the inactivation of its signaling receptor (gp130), reduces the aggressiveness of breast cancer cells in a variety of assays [10]. Blocking antibodies against IL-6, or against its receptor, are in different stages of clinical development. In fact, anti-IL-6 antibodies have been recently approved to treat multicentric Castleman's Disease, a rare lymphoproliferative syndrome [11].

The aim of this study was to establish a collection of breast cancer PDXs that retain the immunohistochemical and molecular characteristics of the original tumor, to assess the relationship between the engraftment of the tumors and the outcome of breast cancer PDXs, to analyze the effect of anti-IL-6 antibodies on their growth and develop an assay to select tumors that depend on IL-6 signaling to grow.

## RESULTS

### Establishment and characterization of a collection of breast cancer PDXs

Out of the 137 samples implanted, 17 breast cancer xenografts were successfully established (12.4%). Successful engraftment rates were correlated with the status of hormone receptors, grade, histology, Ki-67 proliferation index, stage, molecular subtype, neoadjuvant treatments and BRCA status (Table 1). There was also no correlation between engraftment and HER2 positivity, but when we grouped the HER2 positive patients (luminal B HER2+ and HER2+) then the molecular subtype was predictor of engraftment (*p* < 0.04) (data not shown).

**Figure 1:**
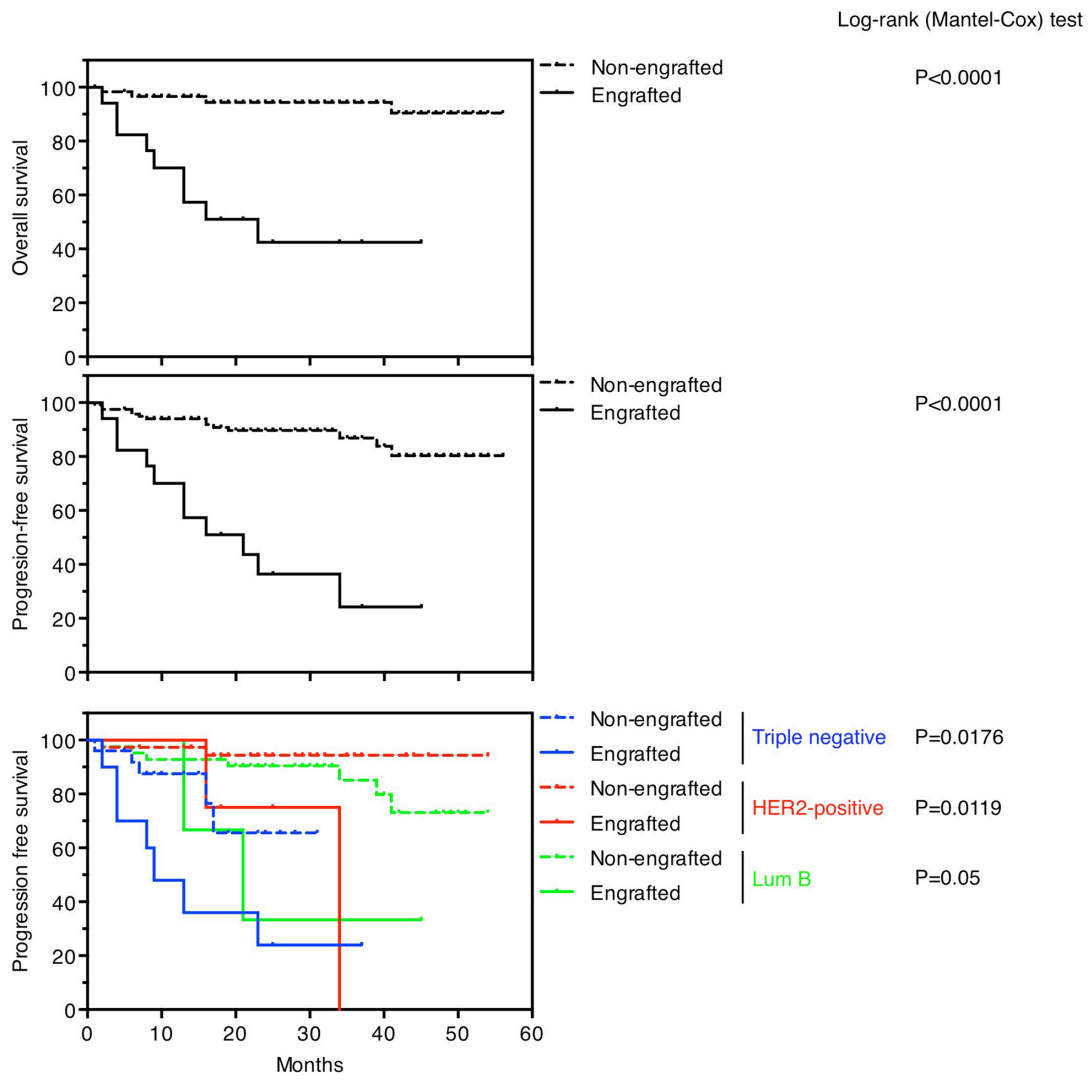
Survival outcomes in patients based on PDXs engraftment Overall survival (upper panel) and progression-free survival (medium panel) in patients whose tumors engrafted versus not. Progression-free survival (lower panel) in patients whose tumors engrafted versus not, according to their intrinsic subtypes.

Successful engraftment was indicator of reduced overall survival and progression free survival (*p* < 0.0001) (Figure 1). Sixteen patients (11.8%) died from breast cancer, of which 9 (56.3%) were successfully engrafted. One hundred and eight patients (78,8%) remained with no evidence of disease, of which 102 (94.4%) failed to generate a PDX. The statistically significant bad prognosis of tumors that successfully engrafted was consistent in three subtypes of tumors analyzed (triple negative, HER2-positive and luminal B) (Figure 1). Thus, in agreement with previous reports [4,6], our collection of breast cancer PDXs is enriched in aggressive tumors of poor prognosis, making it a useful tool to characterize therapies against the most deadly tumors.

### Effectiveness of anti-IL-6 therapy on the growth of PDXs

IL-6 promotes the growth of breast cancers [12,13]. We have recently shown that the autocrine production of IL-6 by naturally occurring senescent cells fosters growth of a HER2-positive tumor. Accordingly, this PDX (referred to as PDX118 in the present manuscript) is sensitive to anti-IL-6 blocking antibodies [14].

To extend this observation we used five additional PDX models. In addition to another HER2-positive PDX, we selected four triple negative PDXs, we chose this subtype because it has been shown that it also depends on IL-6 signaling to grow [15] and it is the only subtype of breast cancer without targeted therapy.

**Figure 2:**
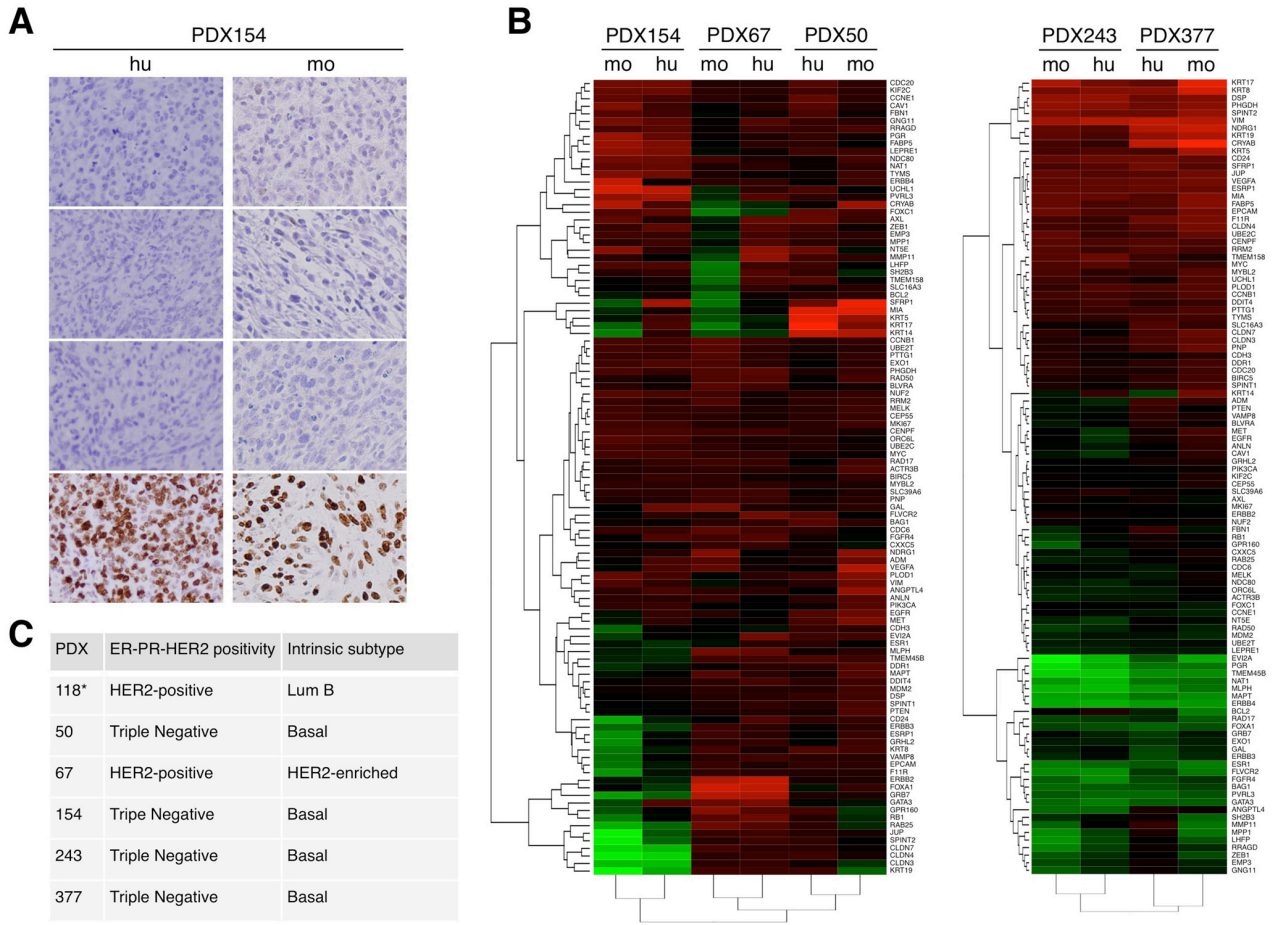
Characterization of different breast cancer PDXs **A.** The expression of the estrogen receptor (ER), progesterone receptor (PR), human epidermal growth factor receptor 2 (HER2) and Ki-67 were evaluated in samples from the indicated patient (hu, human) and the corresponding PDX (mo, mouse). **B.** Unsupervised hierarchical clustering of the samples from the original tumor (hu) or samples from the corresponding PDXs (mo) according to the levels of expression of 110 selected genes analyzed using the Counter platform. All tumors were assigned to an intrinsic molecular type of breast cancer (Luminal A, Luminal B, HER2-enriched, and Basal-like) [26]. The analyses of PDXs 154, 67 and 50 and PDXs 243 and 377 are presented separately because they were performed in different experiments. C. Results of analyses performed as in A and B on the indicated PDXs. Note that the characterization of PDX118 has been published elsewhere [16].

The classification of the different PDXs was assessed by analyzing the expression of hormone receptors (ER and PR) and HER2 (Figure 2A, 2C (see also supplementary Figure S1)). In addition, we determined intrinsic subtypes of breast cancer according to the levels of expression of selected genes. In accordance with the results of the immunohistochemical analysis, PDX50, 154, 243 and 377 were classified as basal, PDX67 was HER2-enriched (Figure 2B, 2C) and PDX118 was luminal B [16]. Supporting the close resemblance between original tumors and PDXs, samples from each tumor clustered with their corresponding PDX models (Figure 2B).

**Figure 3:**
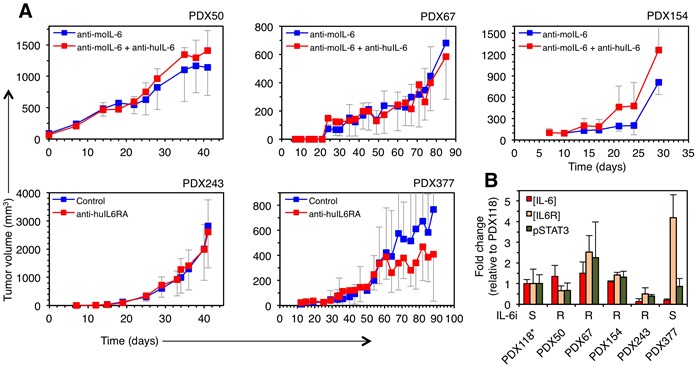
Effect of therapies against IL-6 signaling on the growth of different breast cancer PDXs **A.** PDXs were orthotopically implanted into NOD/SCID mice and treated with two anti-IL-6 signaling therapies (anti-IL-6 or anti-IL6RA blocking antibodies). Note that, when using anti-IL-6, in order to analyze the role of autocrine IL-6, PDXs were treated with anti-mouse IL-6, which does not cross-react with human IL-6 and, thus, does not affect the IL-6 produced by human tumor cells. Anti-IL-6RA antibody does not recognize mouse IL-6 receptor [27]. Tumor volumes were determined at the indicated time points and expressed as averages. The sensitivity of PDX118 to inhibition of IL-6 signaling has been determined elsewhere [14]. **B.** Basal levels of IL-6, IL-6RA and phospho-STAT3 were determined in lysates from the indicated PDX tumors. The results are expressed as averages of three independent tumors ± SD.

To analyze the effect on tumor growth of inhibiting IL-6 signaling, we used two alternative therapies: anti-IL-6 and anti-IL6RA blocking antibodies. Since the only specific receptor of IL-6 is IL6Ralpha, the use of blocking antibodies against the cytokine or its cognate receptor should be, in principle, functionally equivalent. Thus, we used antibodies targeting indistinctly these components. Only one of the PDXs (PDX377) showed a tendency to respond to the inhibition of IL-6 signaling (Figure 3A), although the difference did not reach statistical significance. This result strongly suggests that only some breast cancers respond to anti-IL-6 therapies, underscoring the need of identifying the sensitive tumors, in order to save unnecessary treatments.

Analysis of the levels of IL-6 and IL6RA showed no significant differences between the sensitive and insensitive PDXs (Figure 3B), indicating that the levels of expression of these components do not correlate with sensitivity to anti-IL-6 therapy. Further, the basal levels of phospho-STAT3, the active form of one of the main intracellular signals transduced by IL-6 and other cytokines [17], did not correlate with responsiveness to anti-IL-6 or anti-IL-6RA blocking antibodies (Figure 3). Thus, the efficacy of anti-IL-6 therapy cannot be predicted by analyzing the absolute levels of the cytokine, its cognate receptor or one of its signal transducers.

### The efficacy of anti-IL-6 therapies can be gauged by functionally analyzing phospho-STAT3

The STAT3 pathway fosters the growth of different tumors including those of the breast. Since, in addition to IL-6, several cytokines and agonists of G-protein-coupled receptors (GPCRs) and Toll-like receptors (TLRs) can activate STAT3 [17], we reasoned that only tumors in which the activation of STAT3 depends on IL-6 will respond to therapies blocking this cytokine. Thus, we aimed to establish a functional assay to determine the dependency of STAT3 signaling from IL-6.

**Figure 4:**
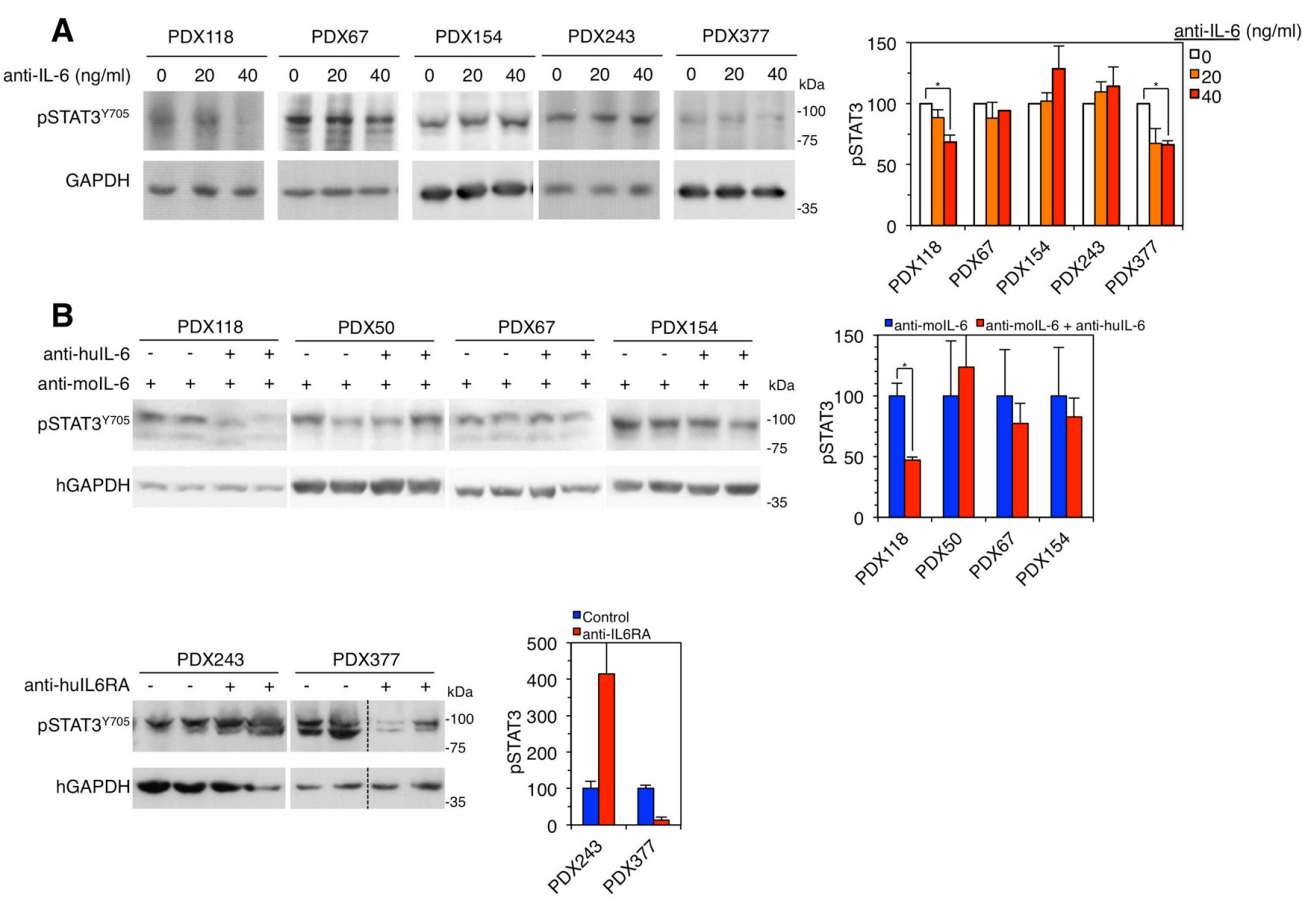
Effect of anti-IL-6 on the levels of phospho-STAT3 **A.** Left, cell cultures established from the indicated PDXs were treated with different concentrations of anti-IL-6. Then, cells were lysed and the cell lysates were analyzed by Western blot with the indicated antibodies. Right, results from four independent experiments were quantified and expressed as averages ± SD. * *P* < 0.05 by the Student's t test. **B.** Left, samples from the indicated PDXs treated as shown in Figure 3 were lysed and analyzed by Western blot with the indicated antibodies. Right, results were quantified and expressed as averages ± SD. * *P* < 0.05 by the Student's t test.

Establishing cell cultures directly from human breast cancer samples is laborious and inefficient. In addition, culture conditions impose a highly selective pressure, which results in the expansion of cells particularly fitted to grow in vitro. As a consequence much of the original intratumoral heterogeneity is lost. In contrast, PDXs retain much of this heterogeneity and because of the availability of large amounts of tumor material, establishing low-passage cell cultures is achieved readily. We succeeded establishing cultures from 5 out of the 6 PDXs and analyzed in vitro the ability of anti-IL-6 to interfere with STAT3 activation. Only in cultures from PDXs 118 and 377, the blocking antibody against IL-6 impaired the activation of STAT3 (Figure 4A) indicating that, despite expressing detectable levels of IL-6 (Figure 3B), in PDXs 67 and 154 alternative factors activate this signaling pathway.

To confirm this conclusion, we analyzed the levels of phospho-STAT3 in samples from the PDXs treated as shown in Figure 3A. Confirming the results obtained with in vitro cultures, only in the PDX sensitive to anti-IL-6 signaling therapies, the levels of phospho-STAT3 decreased with treatment (Figure 4B). In the rest of PDXs, autocrine factors maintained or even increased the activation of STAT3.

These results show that a functional assay should be used to identify breast tumors sensitive to anti-IL-6 therapy.

## DISCUSSION

Accumulating evidence show the contribution of IL-6 to the progression of breast cancers. IL-6 regulates normal stem cell self-renewal in the breast and induces malignancy in stem cells from human breast carcinoma [18]. In addition, IL-6 cooperates with HER2 to promote breast cancer [19] and may be involved in the resistance of HER2-positive tumors to anti-HER2 therapy [12]. A major source of IL-6 in breast tumors are naturally occurring senescent cells; inhibition of IL-6 from this source impairs the growth of a PDX [14]. Thus, it has been suggested that anti-IL-6 therapies, alone or in combination with other therapies, may be efficacious to treat breast cancers [20].

Signals transduced by STAT3 are required for the progression of different cancers, including those of the breast (recently reviewed in [17].). Most of the effects of IL-6 are thought to be exerted through STAT3 but additional factors regulate this signal transducer. Therefore, in order to identify tumors sensitive to anti-IL-6 therapies, it is important to develop functional assays to determine in which tumors STAT3 is primarily regulated by IL-6.

PDXs recapitulate the architecture as well as the molecular and cellular heterogeneity of the tumors from which they are derived [1, 21]. In agreement with this view, the analysis of the expression of a selected group of genes shows the similarity of the PDXs with the tumors from which they were derived (Figure 2 and supplementary Figure S1). The reported efficiency of successful engraftments of breast cancers in immunodeficient mice are highly variable but compared with other tumors, is generally low (12-40%) [1,4,6,22,23]. In agreement with previous reports, successful engraftment was an independent predictor for overall survival and poor outcome (Figure 1). Tumors with high proliferation index, higher grade and more aggressive behavior have higher engraftment rates. In addition, neoadjuvant chemotherapy is an independent factor for successful engraftment, probably due to the higher aggressiveness of these tumors, which responded partially to the treatment, allowing us to obtain tissue. Thus, highly aggressive breast tumors tend to be overrepresented in PDX collections, advising the use of this preclinical model to test novel therapies or combinations against life threatening tumors.

Using these models, we have tested the anti-tumor efficacy of anti-IL-6 blocking antibodies on patient derived xenografts (PDXs) (this report and [14]). The fact that four out of six PDXs did not respond to anti-IL-6 antibodies (Figure 3) highlights the need for reliable biomarkers of sensitivity for this treatment. We found no significant differences in the levels of IL-6, its cognate receptor or phospho-STAT3 in samples from the PDXs analyzed (Figure 3B), showing that the levels of these components do not predict the response to anti-IL-6 therapy. We hypothesized that only those tumors in which the activation of STAT3 relies on IL-6, will respond to therapies directed against this cytokine. The analysis of cultures obtained from three PDXs and the analysis of phospho-STAT3 levels in the PDXs treated with anti-IL-6 confirmed this hypothesis (Figure 4A).

In summary, our data suggests that only breast tumors in which the activation of STAT3 is dependent on IL-6 will be sensitive to therapies against this cytokine. Since STAT3 can be activated by a variety of cytokines, including IL-11, CNTF, LIF and G-CSF [17], in breast cancer, it is crucial to identify those tumors that depend on IL-6. The use of PDXs, and the cell cultures derived from them, is a laborious but feasible procedure to identify them.

## MATERIALS AND METHODS

### Ethics statement

Investigation has been conducted in accordance with the ethical standards and according to the Declaration of Helsinki and according to national and international guidelines and has been approved by the authors' institutional review board.

### Patient and tumor material

A total of 137 breast cancer specimens were obtained from the operation room and transferred to the pathology department where the breast cancer samples were collected, transferred to the animal facility and implanted into the mice. All the samples were implanted during the following 60-90 minutes after surgical removal. All patients have signed an informed consent and the study was approved by the Ethics Committee of the Vall d'Hebron Hospital.

Of samples collected, 136 were primary tumors and 1 was an axillary metastasis. The median age was 60 (range, 24-92). Regarding tumor characteristics, most of the tumors were invasive ductal carcinoma (92.7%), grade III (59.1%), ER positive (68.6%), PR negative (52.5%), HER2 negative (69.3%) and Ki-67 > 20% (80.2%). Half of the patients (53.9%) were stage II, while 6 (4.3%) were stage IV (Table I).

The proportions of tumors classified by molecular subtypes using immunohistochemistry were 10.2% luminal A (14 patients), 33.6% luminal B HER2 negative (46 patients), 26.3% luminal B HER2 positive (36 patients), 4.4% HER2 positive (6 patients) and 25.5% triple negative (35 patients).

The majority of patients (78.1%) did not receive any treatment before surgery, while 21.2% were treated with chemotherapy. Six patients (4.4%) were BRCA mutation carriers.

Median time to implantation was 3.6 months (range 1-6). Triple negative tumors grew faster as tumor grafts (median time to detection ∼2.4 months) than the rest (median time to detection ∼5 months), in agreement with the clinical situation. Engraftment rates in correlation with tumor characteristics are summarized in Table 1.

**Table 1:** Clinical characteristics and corresponding engraftment rates

Tumor Characteristics	Engraftment success		*P* value
	*n* (%)		
	No	Yes	
Histology			
IDC	113 (89)	14 (11)	0.006
ILC	4 (100)	0 (0)	
Metaplasic	0 (0)	3 (100)	
Others	3 (100)	0 (0)	
Grade			
II	55 (98.2)	1 (1.7)	0.001
III	65 (80.2)	16 (19.7)	
Estrogen Receptor			
Negative	32 (74.4)	11 (25.5)	0.004
Positive	88 (93.6)	6 (6.3)	
Progesterone Receptor			
Negative	58 (80.5)	14 (19.4)	0.009
Positive	62 (95.3)	3 (4.6)	
HER2			
Negative	82 (86.3)	13 (13.6)	0.58
Positive	38 (90.4)	4 (9.5)	
Ki-67			
Low (< 20)	27 (100)	0 (0)	0.02
High (> 20)	93 (84.5)	17 (15.4)	
Subtype			
Luminal A	14 (100)	0 (0)	0.020
Luminal B HER2 -	43 (93.4)	3 (6.5)	
Luminal B HER2 +	33 (91.6)	3 (8.3)	
HER2 +	5 (83.3)	1 (16.6)	
Triple negative	25 (71.4)	10 (28.5)	
Stage			
IA	29 (100)	0(0)	0.04
IB	3 (75)	1 (25)	
IIA	37 (88.1)	5 (11.9)	
IIB	28 (87.5)	4 (12.5)	
III	19 (79.1)	5 (20.8)	
IV	4 (66.6)	2 (33.3)	
BRCA			
No	118 (90.08)	13 (9.9)	0.002
Yes	2 (33.3)	4 (66.6)	
Neoadjuvant Treatment			
NO	99 (92.5)	8 (7.4)	0.002
YES	20 (68.9)	9 (31.03)	

### Generation of breast cancer patient-derived xenografts and treatments

All the samples were kept and transported in DMEM/F12 with 5% FBS (DMEM5) (Invitrogen). Three different methods were used to perform the implantation: fragments, organoids and single cells. To implant fragments, samples were cut in 3 mm^3^ pieces and implanted readily. For organoids and single cells, tissue was minced into 1-2 mm^3^ pieces in a 10 cm Petri dish on ice and digested during 1-4 hours (the time was adjusted depending on the sample size) in DMEM5 with 2mg/ml collagenase IA, at 37°C in a rotating wheel. The procedure was finished when the large pieces of tumor disappeared. The solution of digested tumor was diluted 3 times in DMEM5 and non-totally digested tissue pieces were removed by decantation. The supernatant was serially centrifuged to obtain 3 different fractions: fraction A was enriched in organoids, fraction B in single epithelial cells, stromal cells and blood cells and fraction C in fibroblasts. Organoids from fraction A were injected with Matrigel in a 1:1 mixture. The cell suspension resulting from mixing organoid-depleted fraction A and fraction B was depleted from leukocytes, endothelial cells, mesenchymal cells, erythroid precursors and macrophages with antibodies against CD45, CD31, CD140b, CD235a and CD16 and injected with Matrigel.

In all cases, fragments, organoids or single cells were implanted in the number four fat pad of six to eight-week old NOD.CB17PrkdcSCID/J (NOD/SCID) mice (Charles River). To do this, mice were anesthetized and shaved, the fourth and fifth sets of nipples were localized and an inverted Y incision from the midline point between the fourth set of nipples was made, ending between the fourth and fifth sets to expose the fourth and fifth fat pads on one side. After the injection, the animal was sutured and analgesics injected. Animals were kept in a clean cage with 1 uM 17-β-estradiol-supplemented drinking water. Tumor xenografts were measured with callipers every 3 days and tumor volume was determined using the formula: (length x width2) x (pi/6). At the end of the experiment, the animals were anesthetized with a 1.5 % isofluorane-air mixture and were sacrificed by cervical dislocation.

Anti-human IL-6 (CNT328/Siltuximab, 20 mg/kg in sterile PBS) and anti-mouse IL-6 (same dose) were given intraperitoneally once weekly from day 0. In the case of anti-IL6RA treatment, animals were randomized when tumors reached 150 mm^3^ into control (sterile PBS) and treated (tocilizumab, 100 ug per mouse in sterile PBS) groups, and treatments were administered intraperitoneally 3 times per week.

Mice were maintained and treated in accordance with institutional guidelines of Vall d'Hebron University Hospital Care and Use Committee.

### Immunohistochemical and molecular characterization of breast cancers and PDXs

Tumor xenografts were removed, fixed in 4% buffered formaldehyde for 24 h and then paraffin-embedded (FFPE). Sequential 5-um-thick slices were then obtained and immunostained for estrogen receptor, progesterone receptor, c-erbB2 or Ki-67 (Dako). The same procedure was used for human breast cancer samples. Diagnosis and histopathological characteristics were confirmed by a pathologist.

For each sample, three 2-mm cores enriched with tumor tissue were obtained from FFPE tumor blocks. RNA was purified using the High Pure FFPE RNA Micro Kit (Roche Applied Science) and 100 ng of total RNA was used for the Nanostring n-Counter platform to measure the expression of 110 selected genes. nCounter raw data was log base 2 transformed and normalized using 5 house-keeping genes (ACTB, MRPL19, PSMC4, RPLP0 and SF3A1). All tumors were assigned to an intrinsic molecular subtype of breast cancer (Luminal A, Luminal B, HER2-enriched and Basal-like) or the Normal-like breast group using the expression of the 50 PAM50 genes and the PAM50 subtype predictor algorithm from Parker et al [24].

### Cell culture from PDXs

Tumors were disaggregated with 2 mg/ml collagenase IA in DMEM5 and 1×105 cells were plated over night in six-well plates. Next day, tocilizumab was added and cells were harvested and lysed 24 hours later.

### Enzyme-linked ImmunoSorbent assay

Concentration of IL-6 and IL-6R alpha was determined in tumor cell lysates according to the manufacturer's instructions.

### Protein extraction and immunoblotting

They were performed as described in [25]. Briefly, cells were washed twice with ice-cold 1X PBS and proteins were extracted with 20 mM Tris-HCl pH 7.4, 137 mM NaCl, 2 mM EDTA, 10% glycerol, 1% Nonidet P-40 supplemented with 50 mg/ml leupeptin, 50 mg/ml aprotinin, 0.5 mM sodium orthovanadate and 1 mM phenylmethylsulfonyl fluoride (Sigma). Breast tumor samples were homogenized in the same lysis buffer with a Polytron homogeneizer. Protein extracts were quantified using bicinchoninic acid protein assay reagent (Pierce), resolved by SDS-polyacrylamide gel electrophoresis and transferred to nitrocellulose membranes. Primary antibodies recognized human specific forms of GAPDH (Abcam) and phospho-STAT3 (Y705) (R&D). Secondary antibodies included horseradish peroxidase-linked anti-rabbit IgG and anti-mouse IgG (Amersham GE Healthcare). Proteins were detected with Immobilon western chemiluminescent HRP substrate (Millipore). Signals in Western blots were quantified with the software ImageJ 1.38 (NIH, Bethesda, MD, USA).

### Statistical analysis

Patient clinical and pathologic tumor characteristics were collected. To determine whether the ability of a tumor to generate a xenograft might be an indicator of patient prognosis, graft data and clinical outcome information was also gathered. Results were considered to be statistically significant at *P* < 0.05

## SUPPLEMENTARY MATERIALS FIGURE


