# Supplementary material for: Modeling anti-IL-6 therapy using breast cancer patient-derived xenografts
Source: Oncotarget. 2016 Sep 1;7(42):67956–65. doi: 10.18632/oncotarget.11815 (PMC5356531; doi:10.18632/oncotarget.11815)
Supplement: Supplementary file 1 [file oncotarget-07-67956-s001.pdf]

Modeling anti-IL-6 therapy using breast cancer patient-derived xenografts

Supplementary Material

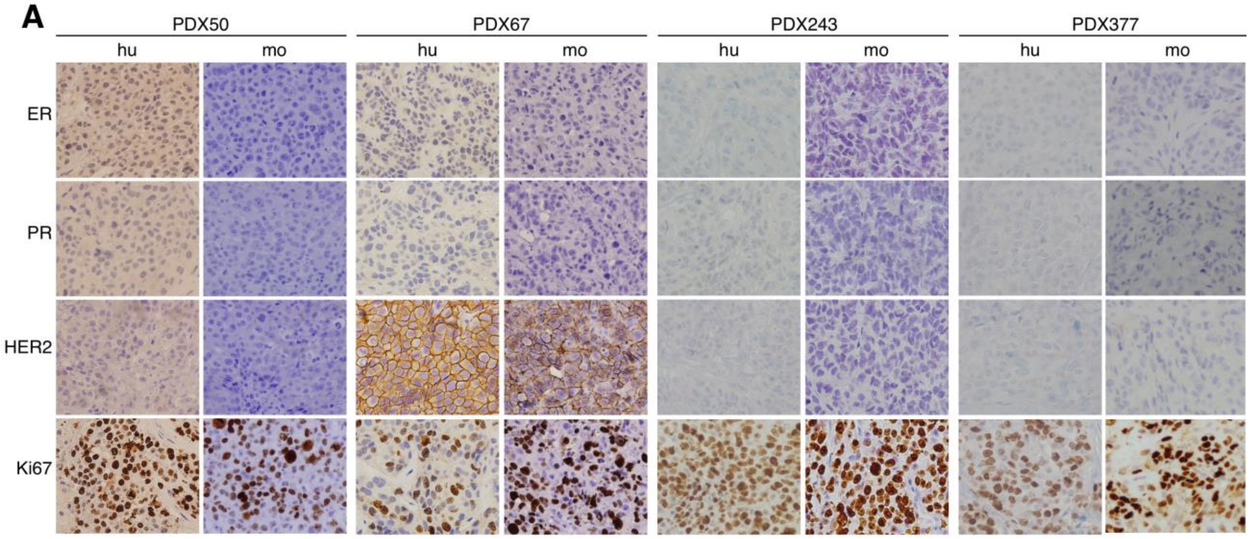

Fig. S1. The expression of the estrogen receptor (ER), progesterone receptor (PR), human epidermal growth factor receptor 2 (HER2) and Ki-67 were evaluated in samples from the indicated patient (hu, human) and the corresponding PDX (mo, mouse).
